# Long-Term Post-Stroke Changes Include Myelin Loss, Specific Deficits in Sensory and Motor Behaviors and Complex Cognitive Impairment Detected Using Active Place Avoidance

**DOI:** 10.1371/journal.pone.0057503

**Published:** 2013-03-07

**Authors:** Jin Zhou, Jian Zhuang, Jie Li, Evelyn Ooi, Jonathan Bloom, Carrie Poon, Daniel Lax, Daniel M. Rosenbaum, Frank C. Barone

**Affiliations:** 1 Department of Neurology, State University of New York Downstate Medical Center, Brooklyn, New York, United States of America; 2 Robert F. Furchgott Foundation, State University of New York Downstate Medical Center, Brooklyn, New York, United States of America; 3 Department of Physiology and Pharmacology, State University of New York Downstate Medical Center, Brooklyn, New York, United States of America; Université Pierre et Marie Curie, France

## Abstract

Persistent neurobehavioral deficits and brain changes need validation for brain restoration. Two hours middle cerebral artery occlusion (tMCAO) or sham surgery was performed in male Sprague-Dawley rats. Neurobehavioral and cognitive deficits were measured over 10 weeks included: (1) sensory, motor, beam balance, reflex/abnormal responses, hindlimb placement, forepaw foot fault and cylinder placement tests, and (2) complex active place avoidance learning (APA) and simple passive avoidance retention (PA). Electroretinogram (ERG), hemispheric loss (infarction), hippocampus CA1 neuronal loss and myelin (Luxol Fast Blue) staining in several fiber tracts were also measured. In comparison to Sham surgery, tMCAO surgery produced significant deficits in all behavioral tests except reflex/abnormal responses. Acute, short lived deficits following tMCAO were observed for forelimb foot fault and forelimb cylinder placement. Persistent, sustained deficits for the whole 10 weeks were exhibited for motor (p<0.001), sensory (p<0.001), beam balance performance (p<0.01) and hindlimb placement behavior (p<0.01). tMCAO produced much greater and prolonged cognitive deficits in APA learning (maximum on last trial of 604±83% change, p<0.05) but only a small, comparative effect on PA retention. Hemispheric loss/atrophy was measured 10 weeks after tMCAO and cross-validated by two methods (e.g., almost identical % ischemic hemispheric loss of 33.4±3.5% for H&E and of 34.2±3.5% for TTC staining). No visual dysfunction by ERG and no hippocampus neuronal loss were detected after tMCAO. Fiber tract damage measured by Luxol Fast Blue myelin staining intensity was significant (p<0.01) in the external capsule and striatum but not in corpus callosum and anterior commissure. In summary, persistent neurobehavioral deficits were validated as important endpoints for stroke restorative research in the future. Fiber myelin loss appears to contribute to these long term behavioral dysfunctions and can be important for cognitive behavioral control necessary for complex APA learning.

## Introduction

Stroke is the 4th leading cause of death and a leading cause of disability in the United States (http://www.strokeassociation.org/STROKEORG/AboutStroke/About-Stroke_UCM_308529_SubHomePage.jsp). The consequences of stroke include significant deficits in both sensory-motor and cognitive functioning. The cost of rehabilitation and care in 2010 was approximately: $273 Billion per year. However, by 2030, increased cerebrovascular disease is expected to result in a tripling of stroke medical costs to $818 Billion per year. Dementia is expected to dramatically increase in the future related to the continued aging of the world population. Not only is cognitive decline and dementia associated with increased stroke risk (e.g., vascular cognitive impairment) [1], [2], but the absolute risk for dementia is dramatically increased within the first few months following a stroke (e.g., post-stroke vascular dementia) [3].

Measurements of functional-neurological outcome after experimental stroke-induced brain injury have been made for years but no effective therapy is available to improve post-stroke functional outcome in humans [4]. In order to accurately reflect the properties of the human clinical situation, experimental stroke research approaches attempt to identify neurobehavioral-neurological deficits, such as post-stroke sensorimotor deficits [5] and cognitive impairments [6]. Unilateral middle cerebral artery occlusion (MCAO), a significant cause of ischemic stroke in both humans and rodents, produces contralateral neurological-sensor-motor deficits and a compensatory reliance on the less impaired side of the body ipsilateral to the injured brain. The most commonly used methods to monitor these sensorimotor functions include the Modified Neurological Severity Score (mNSS; a compendium of neurological neurobehavioral tests), hindlimb placement, and forelimb foot fault and forelimb cylinder placement behaviors [5], [7], [8], [9]. Systematic measurements of individual behavior functions that can be used in long-term outcome studies are not yet available.

Deficits in cognitive performance following experimental stroke can be problematic because of these sensory-motor impairments. They can confound interpretation of the cognition data. Several different protocols have been used to assess cognitive function in rats subjected to MCAO including active avoidance [10], passive avoidance [11], the radial arm maze [12], and the Morris water maze [13], [14].

Even under the best testing conditions cognition is the result of many processes and is highly variable. Passive avoidance (PA) has most often revealed MCAO-induced cognitive deficits [10], [11]. However, PA performance following MCAO does not always correlate with changes in histological outcome [15]. Although active avoidance paradigms have been utilized for decades, the active place avoidance (APA) paradigm is relatively new. In APA learning rats learn to avoid a stationary shock zone on a rotating arena [16], [17], [18]. APA is relatively complex in that optimal performance requires perceptual segregation since rats must attend to distal, stationary visual cues to identify the location of the shock zone while ignoring irrelevant proximal olfactory cues on the rotating arena [18]. The task places a high cognitive demand on rats and is very sensitive to unilateral traumatic brain injury [16], [19], [20]. However, it has never been used to evaluate complex cognition following MCAO-induced unilateral ischemic stroke brain damage.

In experimental stroke research, histological analysis is usually performed to evaluate post-stroke damage providing measurements of infarct size [9]. However, degree of infarction measurements do not provide information on MCAO-induced damage to fiber tracts/axons, or cellular injury in remote areas important in cognition such as the hippocampus or in areas adjacent to the stroke-induced infarction (i.e., peri-infarct areas). Certainly the profiling of many different sensory, motor and cognitive behaviors will provide increased opportunity to better understand brain protection and restoration of function in the future. These issues can account for the inconsistent and poorly correlated degree of infarction to severity of neurological deficits seen previously [21].

In the present study, many long-term neurological-cognitive behaviors were profiled over time following 2 hr MCAO followed by reperfusion (tMCAO) compared to Sham Surgery. Brain morphological measurements included degree of infarction, the integrity of the most vulnerable neural cell layer in the hippocampus and myelin loss in several major forebrain fiber tracts. Our purpose was to establish a paradigm to better understanding the consequences of stroke and apply these new approaches to our future research. The identification of persistent neurological and cognitive deficits associated with brain changes, including injury to fiber tract systems that are essential to complex cognitive performance, are expected to provide us with a framework for investigating treatment approaches that facilitate brain restoration (i.e., reorganization, synaptic plasticity and recovery of lost function) post-stroke.

## Materials and Methods

### 1. Ethics Statement

All animal protocols utilized in this work are approved by the Animal Care and Use Committee of the State University of New York at Downstate Medical Center.

### 2. Animals and Surgical Procedures

Male Sprague-Dawley rats weighing 250 to 300 g from Charles River Laboratories (Wilmington, MA, USA) were housed in pairs under controlled laboratory conditions (e.g., 12 h light/dark cycle, controlled temperature and humidity, ad lib access to food and water). Briefly, under isoflurane anesthesia, a 3-0 monofilament suture (Ethicon, Somerville, NJ, USA) with a heat-blunted tip and a coat of poly-L-lysine (Sigma-Aldrich, St Louis, MO, USA) was inserted through the proximal external carotid artery, advanced into the internal carotid artery and positioned to occlude the origin of middle cerebral artery (i.e., 18 to 20 mm from the common carotid artery bifurcation). Rats recovered from anesthesia but after 2 hours of occlusion rats were re-anesthetized with isoflurane and the intraluminal filament was removed (i.e., the artery was reperfused). Sham surgery animals were subjected to the same procedure but without occluding the artery. Body temperature was continuously maintained at 37.0°C±0.5°C using the Heat Therapy Pump-1500 and Temperature Therapy Pad (Adroit Medical Systems, Louden, TN, USA). Body temperature was maintained until animals completely recovered from anesthesia and displayed normal motor activity. Animals were then placed in their home cages, monitored closely over the next 4 hours and then daily for the rest of the study. Only <5% of tMCAO surgery rats were excluded from the study due to unsuccessful tMCAO identified after reperfusion of the artery when put back in their home cage. The “unsuccessful tMCAO” criteria was: (1) no exhibition of contralateral limb weakness and hemiparalysis with maintained ability to hold their head in a straight position when suspended by their tail or, (2) exhibition of moribund behavior that results from brain hemorrhage due to thread placement through a blood vessel [9].

### 3. Body Weight and Neurological Scores

#### Body Weight

Body weight was monitored before surgery and at day 1 (d1), and at weeks 1, 2, 4, 6 and 10 (w1, w2, w4, w6 and w10, respectively) after surgery using a CS2000 Compact Digital Scale (Ohaus, Parsippany, NJ, USA).

#### Mnss

mNSS evaluation was performed just prior to surgery and at d1, and w1, 2, 4, 6 and 10 after tMCAO as described previously (Zhou et al., 2011) in order to grade post-stroke sensory-motor neurologic deficits.

#### Individual mNSS Deficit Components

mNSS has been used in experimental stroke for years. In this stidy, instead of general mNSS as an overall indicator of neurological deficits, we have divided the mNSS into distinct behavioral components including motor, sensory, beam balance tests and the absence of several reflexes and/or presence of abnormal movements. The detailed methods and scorings for these components are listed in Table 1. The total score from individual deficit tests making up the mNSS and was scaled from 0 to 18 with 0 as normal and maximal deficit score as 18, which reflected combined sensorimotor, beam balance and reflex-abnormal movement functions [8], [9] were individually analyzed in order to systematically determine those deficits that occurred acutely or persisted more chronically. The individual test data (i.e., motor, sensory and beam balance data) were also analyzed and are also presented graphically.

**Table 1:** Components of the Modified Neurological Severity Score (mNSS).

Methods and Observations for Specific Deficits (Total Maximum Deficit Score Possible-18)	Observed Scores	Maximum Scores
Motor	Cumulative	6
**Rat Suspended by tail**		
Normal response-Balanced suspension	**0**	
Inability to suspend contralateral forelimb (Forelimb Flexion)	**1**	
Inability to suspend contralateral hindlimb (Hindlimb Flexion)	**2**	
Head moves >10 degrees off vertical axis within 30 s	**3**	
**Place Rat on floor**		
Normal response-Straight walk	**0**	
Inability to walk straight	**1**	
Circling toward paretic, ipsilateral side (confirm with lifting tail)	**2**	
Falls down to paretic, ipsilateral side	**3**	

Sensory	Additive	2
**Placing Test - Rat is held with contralateral side next to edge of table providing contralateral whisker stimulation**		
Normal response-Forelimb placed on table	**0**	
No forelimb placement in response to contralateral whisker stimulation	**1**	
**Proprioceptive Test - Rat is held with for-paws on a table surface then pushed to contralateral side providing tactile stimulation**		
Normal response-Forepaw resistance to contralateral lateral push	**0**	
No forelimb resistance to contralateral lateral push tactile stimulation.	**1**	

Beam Balance	Cumulative	6
**Rats place on a long 1 inch-width beam for 60 s**		
Normal response is balance with steady posture for >60 s	**0**	
Grasps side of the beam	**1**	
Hugs Beam and 1 limb falls down from beam	**2**	
Hugs Beam and 2 limb falls down from beam, or spins	**3**	
Attempts to balance on beam but falls off from 40–59 s	**4**	
Attempts to balance on beam but falls off from 20–39 s	**5**	
Falls off; no attempt to balance or hang on beam in <20 s	**6**	

Reflexes/Abnormal Movements	Additive	4
**Cotton Swab into ear canal**		
Normal response-Head Shake/Pina Reflex	**0**	
Lack of Pinna Reflex	**1**	
**Cotton Swab touches cornea**		
Normal response-Eye closes/Cornea reflex	**0**	
Lack of Corneal Reflex	**1**	
**Loud hand clap**		
Normal response-Jumping movement/Startle reflex)	**0**	
Lack of Startle Reflex	**1**	
**Observe daily for 5 min**		
Normal response-No unusual motor behavior	**0**	
Seizures, myoclonus, and/or myodystony	**1**	

### 4. Other Tests on Neurological Deficits

#### Hindlimb Placement Test

Hindlimb placement ability, a proprioceptive response to limb manipulation, was performed just prior to surgery and at d1, and at w1, 2, 4, 6 and 10 after tMCAO. Rats were positioned facing away from the edge of a table with the contralateral hindlimb pulled (i.e., down and away from the table edge. The ability to retrieve and place the hindlimb back onto the table was graded. Immediate and complete placing = 0, delayed (>2 s) placing and/or variable placing = 1, and no placing = 2 [22].

#### Forelimb Foot-Fault Test

The forelimb foot-fault, a motor test, was performed just prior to surgery and at d1, and at w1, 2, 4, 6 and 10 after tMCAO. Here rats were tested for forelimb movement dysfunction while walking on elevated metal grids with randomly missing support bars. The horizontal grids were 85.5×26.5×20 cm^3^ with a glass enclosure for observation. With each weight-bearing step, the forelimb can fall or slip between the metal support bars, which was recorded as a foot fault. The total number of forelimb steps and the total number of foot faults were recorded. The percentage of forelimb foot faults to total steps that occurred within 2 min was calculated [23].

#### Forelimb Cylinder Placement Test

Forelimb cylinder placement, a motor test, was measured/scored just prior to surgery and at d1, and on w1, 2, 4, 6 and 10 after tMCAO. Each rat was placed in an upright Plexiglas cylinder open at both ends and measuring 30 cm high by 20 cm in diameter placed open end down on a table (i.e., confining the rat being tested within), The number of each forelimb, or both forelimb placements on the wall of the cylinder was recorded, and the percentage of each limb or both limbs placements to the total amount of placements were calculated [24].

#### PA Retention – Simple Cognition

**Figure 1:**
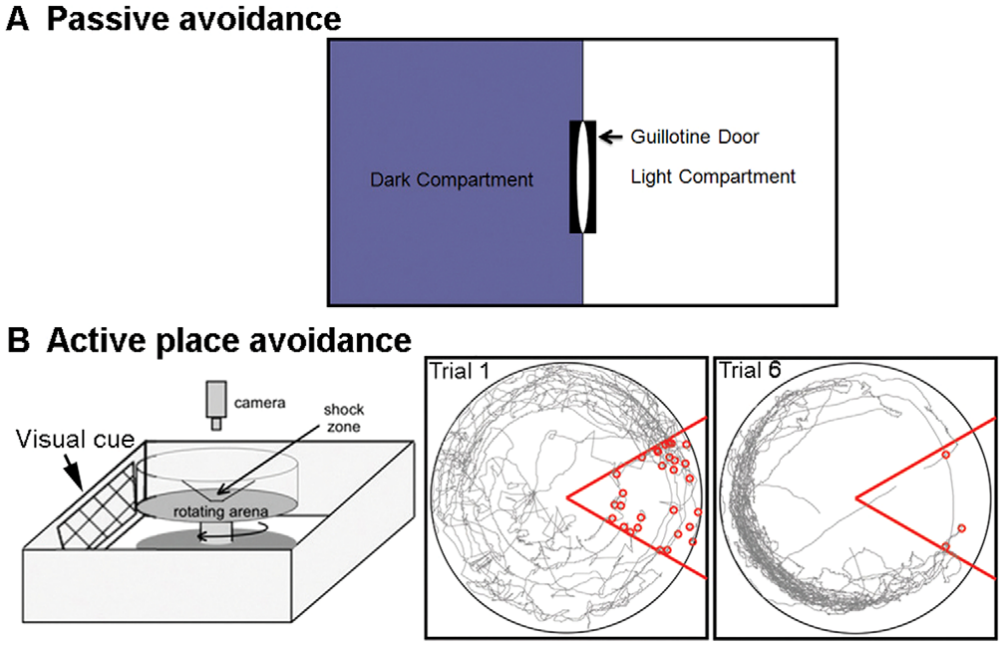
Diagrams and paradigms of PA and APA tests. **A:** A diagram and the paradigm of PACS-30 shuttle box for PA test. The PA PACS-30 shuttle box contains the dark and light compartments separated centrally by an automated guillotine door that prevents inter-compartment movement of the animal. **B:** APA paradigm. LEFT: Schematic representation of the APA apparatus. In the learning phase, rats receive six ten-min open field tests on the rotating arena with a stationary 60° shock zone (angled area). CENTER: A representative computer captured image of the first trial in one learning session. RIGHT: A similar captured image of the sixth trial in the same learning session.

PA is an aversive shock-motivated test classically used to assess the short-term or long-term memory of rats or mice. The PA shuttle box (PACS-30, Columbus Instruments, Ohio) contains two compartments (i.e., dark side and light side) separated centrally by an automated guillotine gate that prevents inter-compartment movement of the animal. The light is located over the door and is always lit on the light side. A shocker grid assembly spans the entire floor area of both compartments. A series of photocells detected the animal's position within the shuttle box (see Figure 1A).

Rats were subjected to three training sessions on day 0 (d0) before surgery and then at w1 and 10 after surgery. Briefly a rat was placed in the light side of PACS 30. The automated guillotine gate was closed and the light side was illuminated at an intensity of 8 (scale of 0-off and 10-brightest provided in PACS 30 software). After an exploration period of 30 s the guillotine gate automatically opens. An entry into the dark side (i.e., the expression of the normal tendency of rats to seek the dark and avoid light) automatically shuts the door and results in punishment of the rat with a single low intensity foot shock (0.5 mA; 5 s). Infrared sensors monitor the transfer from one compartment to another, which was recorded as transfer latency time in seconds (learning acquisition). Each trial lasted for 10 min (see Figure 1A). At baseline before surgery, the number of trials for each rat to learn to completely avoid entry into the dark side (remains in the light side for the full 10 min or 600 s) is recorded as learning acquisition trials. The rats were then divided into two groups as sham and tMCAO group with similar learning capability. After surgery, the rats were tested at w1 and 10. Each test included two consecutive trials. The 1st trial was set up for acquisition with the punishment in the dark compartment and retention was tested by the 2nd trial was given 24 h after the 1st trial, in which the shock was not delivered. The time point of entering the dark compartment on 2^nd^ trial was recorded as latency time [25].

### 5. Cognitive Performances

#### APA Learning – Complex Cognition

In APA rats are placed on a slowly rotating arena (i.e., 1 rotation per min) and receive an aversive stimulus (i.e., electrical shock) when they enter/pass through one stationary quadrant (i.e., the shock zone). The shock zone is eventually sensed and avoided over repeated trials due to the presence of distal, visual cues (i.e., cues outside the arena) [26]. Testing was performed before surgery as the baseline (d0) and at d10 and at w6 after tMCAO. Briefly, it consisted of 6 or 7 ten-min trials (separated by a 10 min rest period) in the slowly rotating behavioral arena during a single day. The arena was placed in an enclosure (i.e., a black ceiling and a black ceiling to floor curtain) with prominent visual landmarks against the surrounding curtain. The rotating arena (an 82-cm diameter metal disc) is surrounded by a 40-cm high transparent wall. Before the test, a subcutaneous shock electrode was implanted in each rat by piercing the skin at the back of the neck with a u-shaped 25-gauge hypodermic needle (i.e., sharp end of the needle was curled to secure it in the skin and an alligator clip was attached from the electrical shock cable to this shock electrode). On each testing day, each rat initially was allowed a 10 min exploration period in the rotating arena. Then a spot-tracker computer program (BioSignal Group, Brooklyn, NY) controlled the shock stimulator. Under these conditions, upon entry of the rat into a constant 60 degree shock zone area, a shock was delivered by passing a constant current (<0.3 mA, 60 Hz, 500 ms) through the cable to the shock electrode through the rat to the grid floor. Entry into the shock zone area was detected by a computer controlled infrared Firewire camera mounted 1.2 m above the arena that recorded the movement of the rat relative to the rotating arena surface. The number of shocks delivered and the total distance traveled during each 10 min trial was recorded. The total distance traveled assessed motor function (see Figure 1B).

### 6. Brain Sectioning and Staining for Measurements of Injury

#### Infarct Measurement

Two different methods were used to measure infarct size that occurred in this work. Both utilized the loss in the ischemic hemisphere as the index of the original degree of infarction [27], [28]. For hematoxylin and eosin staining (H&E), after the final neurobehavioral test rats were perfused fixed and brains were removed and frozen for histological analysis. Brains were sectioned at −17°C on a cryomicrotome. Forebrain coronal sections (30 µM thick) were collected at 600 µm intervals and stained with H&E. Then the slides were scanned and analyzed by image J 1.37c (National Institutes of Health, USA). Total ischemic hemispheric loss was calculated as (right hemisphere – left non-infarct area)/right hemisphere. The data were presented as percentage of right hemisphere. For 2,3,5-triphenyltetrazolium chloride (TTC) staining, brains was removed and cut into 2 mm-thick coronal sections using a brain matrix. The brain sections were then immersed in 1% TTC (Sigma-Aldrich, St. Louis, MO) for 20 min. After scanning, the image was analyzed and calculated using an image analyzing system IMAGE J 1.37v (NIH, Bethesda, MD) by an observer who was blinded to the study. Total ischemic hemispheric loss was calculated as above [9].

#### Measurement of Hippocampal CA1 Neurons

Forebrain coronal sections (30 µm) from perfuse fixed brains were washed with PBS/0.2% TritonX-100 for 3×10 min, and then treated with 1% H_2_O_2_ for 20 min and blocked with 5% normal goat serum for 60 min. Then, the sections were incubated with mouse anti-NeuN monoclonal (primary) antibody (1∶100 in 3%BSA/PBS/0.2% TritonX100, MAB 377; Chemicon International Inc., Temecula, CA) at 4°C overnight. After washing again three times with PBS/0.2% TritonX-100, the sections were applied with the secondary antibody Alex Fluor 647 goat anti-mouse IgG (1∶200, Invitrogen, Carlsbad, CA) in 1% BSA/PBS/0.2% TritonX100 in the dark at 23°C for 1 hour. These immunofluorescent slides were mounted with Prolong Gold Antifade Mounting Medium with 4′, 6-diamidinio-2-phenylindole (DAPI) (Invitrogen, Carlsbad, CA) and observed under an Olympus digital imaging system (Olympus, Center Valley, PA). To exclude any nonspecific staining, consecutive sections were incubated with 3% BSA/PBS/0.2% TritonX100 but omitting the primary antibody as a negative control. Positive fluorescent cells in the hippocampal CA1 zone were counted at 5×20 microscope fields randomly in both ipsilateral and contralateral sides and compared between sham and stroked groups [29].

#### Luxol Fast Blue Staining Of Myelin - White Matter Fiber Tract Injury

Forebrain coronal sections (30 µm) from perfused fixed brains were stained using Luxol fast blue according to protocol provided by the manufacturer (American Mastertech, Lodi, CA). Digital images were collected and the amount of Luxol fast blue staining was assayed using Image J software in regions of interests (ROIs) within external capsule, striatum, corpus callosum and anterior commissure in sections located 0.20 mm–1.70 mm in the coronal plane from Bregma. The optical density from external and internal capsules, the anterior commissure and fiber rich areas within the striatum were measured using an Olympus digital imaging system (Olympus, Center Valley, PA). Measurements were made in hemispheres both contralateral and ipsilateral to the infarct brain area. The intensity signals of external and internal capsules and the anterior commissure in ipsilateral hemisphere were divided by the corresponding intensity signals in contralateral hemisphere and calculated as optical density ratio. In striatum, the ipsilateral positive area divided by the contralateral positive area and was calculated as positive area ratio. The results from tMCAO surgery group were normalized to the sham surgery group and presented as the % optical density ratio in striatum, corpus callosum, external capsule or anterior commissure or % positive area ratio in the striatum [30].

### 7. Post-Stroke Electroretinogram (ERG) - Retinal Function

Visual information is required for APA behavior [26], and common carotid manipulation during tMCAO might produce brief retinal ischemia and modify retinal function [31]. Therefore, visual function needs to be assessed under these conditions. Here, sham and tMCAO surgery rats were evaluated for retinal function using ERG measurements collected and analyzed as described previously [32]. In brief, animals were anesthetized and their pupils were dilated with tropicamide and cyclomydril. Platinum electroencephalographic electrodes were placed on the corneas, and the ERG α-wave and β-wave were identified after presenting full-field stroboscopic flashes at the rate of 1.0/s at a distance of 15 cm. ERG measurements were taken prior to sacrifice for rats in both sham and tMCAO groups at w10. ERG measurements of the right and left eyes were always taken together, with the right eye serving as the non-surgery control and the left eye serving as the surgery eye (i.e., as the potential ischemic eye since the left MCAO was occluded by tMCAO). The degree of the left surgical eye retina response was calculated from the ERG β-wave of the ERG and was normalized to the right nonsurgical (i.e., control) eye using the formula as follows: % β-Wave Amplitude = (ß-wave L/ß-wave R)×100% where R = right eye and L = left eye. The same methods were utilized to provide corresponding α-Wave Amplitudes for comparison.

### 8. Statistical Analysis

For all the experiments, investigators were blinded to surgery treatment during data acquisition and analysis. All data were expressed as means ± S.E.M. For comparison between multiple groups, parametric statistical analyses were performed using one-way or two-way analysis of variances followed by post hoc analyses using the Newman–Keuls test or Bonferroni test for multiple comparisons, respectively. For non-parametric analysis of the data, the Kruskal-Wallis or Friedman Tests was utilized followed by post-hoc analysis using the Mann-Whitney U test. For comparison between only two groups, a student t-test or Mann-Whitney U test was used. Differences between groups were considered significant if P<0.05.

## Results

### 1. Body Weight Changes Were Minor

**Figure 2:**
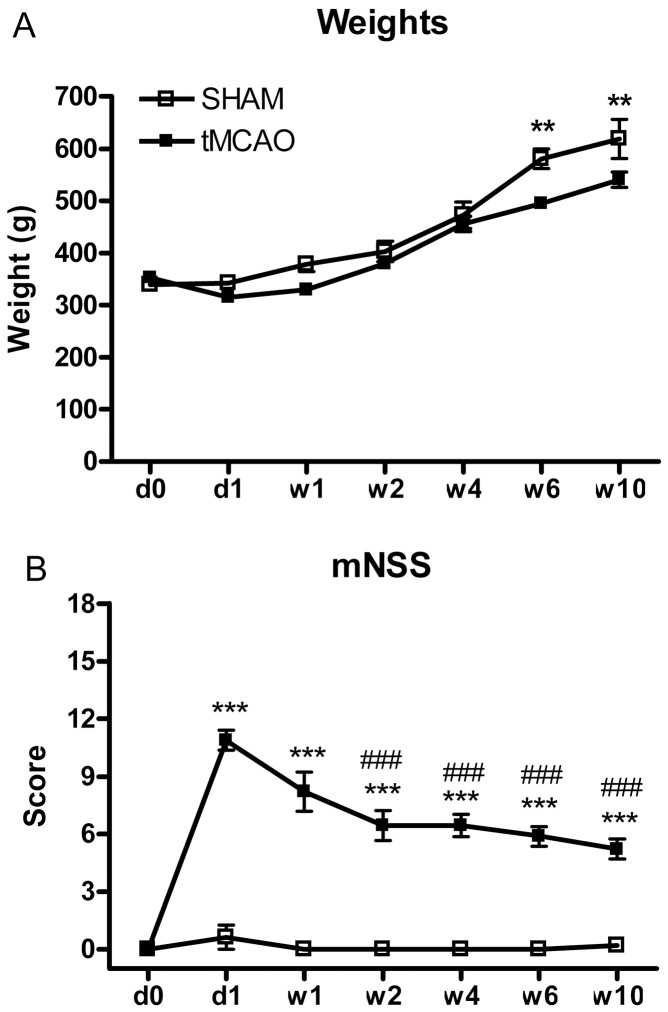
Body weight and composite mNSS score was measured for 10 weeks after tMCAO. The time points for the observation are before the surgery (d0) and at d1 and at w1, w2, w4, w6 and w10 after tMCAO. **A:** tMCAO surgery did affect body weight immediately after surgery. However, tMCAO did result in a small reduction weight gain at w6 and w10 (i.e., failure to gain as much weight as Sham Surgery rats). **B:** mNSS was persistently higher than the control group after stroke for the full 10 weeks of observation. However, there was some spontaneous recovery of function over time (i.e., reduced by about 50% of the original post-stroke deficit at d1). **p<0.01, ***p<0.001 vs. sham group at the corresponding time point. Two-way ANOVA with Bonferonni post-hoc test procedure (n = 8–9 rats/group). ^###^p<0.001 vs. tMCAO group at d1 after stroke. One-way ANOVA with Newman–Keuls test procedure (n = 8–9 rats/group).

Rats were always maintained on free access to food and water before and after surgery. In the first 4 weeks after surgery, there was no significant difference in the body weight between tMCAO and sham surgery groups. However, at w6 and 10 post-surgery, the sham surgery group exhibited an increase in body weight compared to tMACO surgery group. Therefore, although no immediate effects of surgery on body weight observed, longer after surgery only a small increase in body weight occurred in the sham- compared to tMACO-surgery group (Figure 2A).

### 2. Change in mNSS Were Significant and Persistent

When individual components of the neurological deficits are compiled into the total mNSS, the tMCAO group exhibited significant and persistent changes compared to the sham surgery group. The greatest change was observed one day after tMCAO, and although deficits persisted for the 10 week duration of study, the degree of deficit when compared to d1 post-stroke decreased over time from w1 to w10 indicating a significant recovery from the original deficit over time (Figure 2B).

#### Persistent Motor Changes

**Figure 3:**
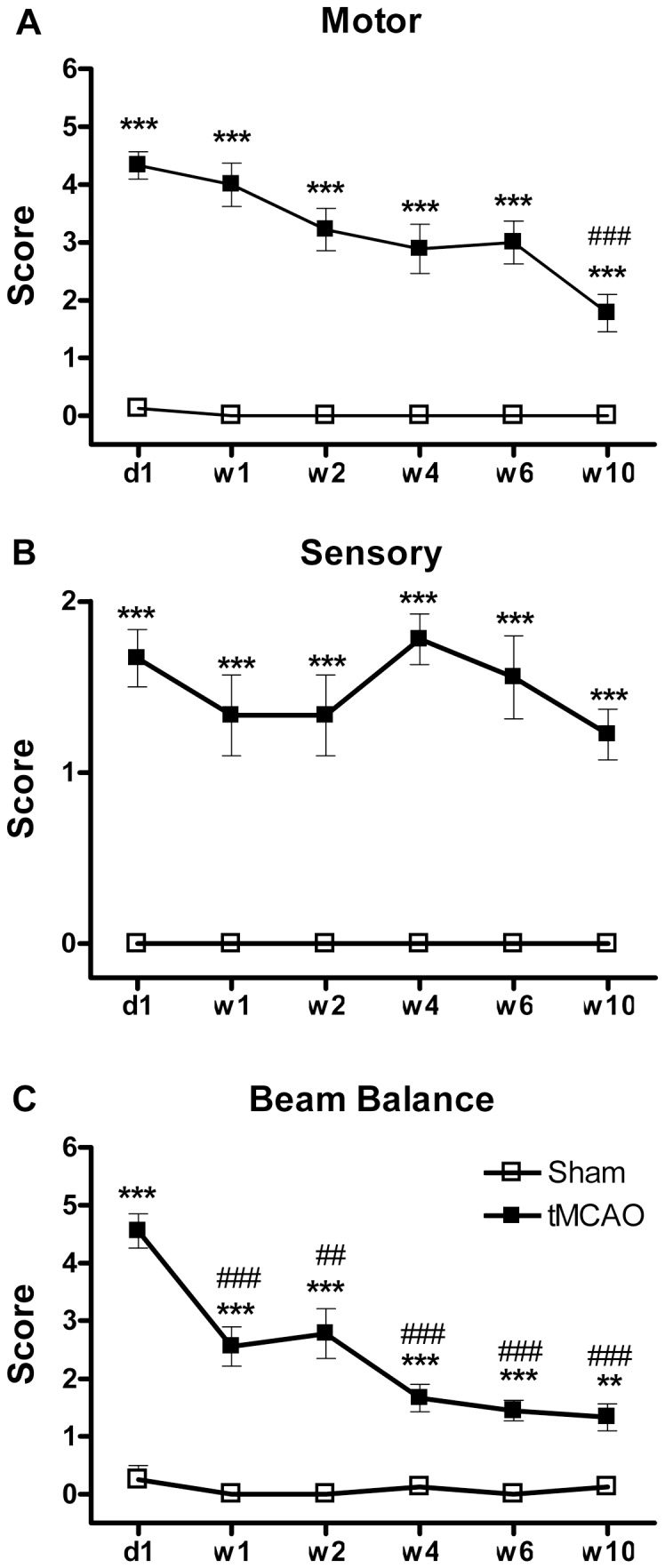
Individual component deficits of the mNSS (See **Table 1**) exhibited persistent changes after tMCAO. **A:** Deficits in motor performance were significant for the full 10 weeks of observation with >50% spontaneous recovery (i.e., decrease from the original d1 deficit) at w10 post-tMCAO. **B:** Deficits in sensory performance were significantly elevated with no spontaneous recovery of function for the full 10 weeks of observation post-tMCAO. **C:** Deficits in beam balance performance were significant for the full 10 weeks of observation with spontaneous recovery of function from the original d1 deficit of ∼40% at w1 and w2 and of ∼70% at w4, w6 and w10 post-tMCAO. **NOTE:** No effects were observed on Reflexes/Abnormal Movements post-tMCAO (data not shown), and thus this series of tests will not be used in future research.

The tMCAO group exhibited significant motor deficits compared to the sham surgery group. The most severe neurological deficits were observed one day after tMCAO, and persisted for the 10 week duration of study. When compared to its own d1 post-tMCAO score, this group showed significant recovery (i.e., a decrease in the severity of deficit) at w10 post-tMCAO (Figure 3A).

#### Persistent Sensory Changes

The tMCAO group exhibited significant sensory deficits compared to the sham surgery group that persisted for the whole 10 week post-tMCAO observation period. When compared to its own d1 post-tMCAO score, this group showed no recovery (i.e., no decrease in the severity of deficit) over the 10 week post-tMCAO observation period (Figure 3B).

#### Persistent Beam Balance Changes

The tMCAO group exhibited significant deficits in beam balance performance compared to the sham surgery group. Maximum deficits occurred at d1 and persisted for the whole 10 post-tMCAO observation period. Compared to d1, the tMCAO did exhibit significant improvement over the 10 week post-tMCAO period, indicating a significant improvement in function (Figure 3C).

#### Reflexes/Abnormal Movements Were Not Affected

tMCAO did not affect pinna, corneal reflexes or the startle response, nor did it produce any abnormal behaviors such as seizures, myoclonus, myodystony or epilepsy, etc in our experiments (data not shown).

### 3. Neurological Deficits Detected by Other Tests

#### Hindlimb Placement Exhibited Persistent Change

**Figure 4:**
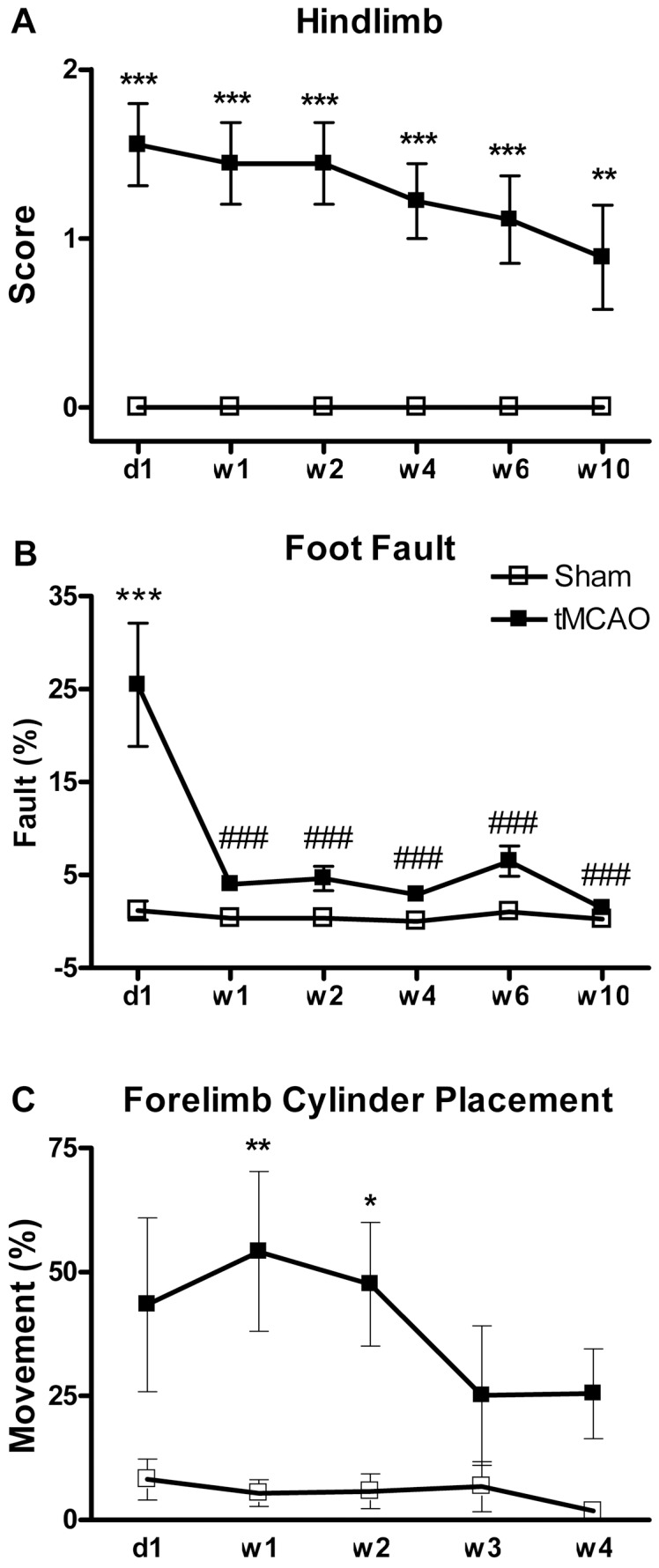
Additional performance tests also exhibited persistent deficits/changes after tMCAO. **A:** Hindlimb Placement performance deficits were significant and exhibited no spontaneous recovery of function (i.e., no decrease from the original d1 deficit) for the full 10 weeks of observation post-tMCAO. **Part B:** Forelimb foot-fault performance deficits were very transient and occurred significantly only at d1 post-tMCAO. **Part C:** Forelimb Cylinder Placement deficits also were transient (but did last longer than foot falut) and persisted only through w2 post-tMCAO. (*p<0.05, **p<0.01 and ***p<0.001 vs. sham group at the corresponding time point, Two-way ANOVA with Bonferonni post-hoc test procedure (n = 8–9 rats/group). ^##^p<0.01 and ^###^p<0.001 vs. tMCAO group at d1 after stroke. One-way ANOVA with Newman–Keuls test procedure (n = 8–9 rats/group).

The tMCAO surgery group exhibited significant deficits in hindlimb proprioception compared to the sham surgery group for the entire observation period of 10 weeks. Compared to d1, the tMCAO group did not exhibit any significant improvement in function, although there was a trend to do so (Figure 4A).

#### Forelimb Foot Fault Exhibited Only Transient Change

The tMCAO group exhibited a significant but very transient forelimb foot fault deficit at only day 1 post-tMCAO. Compared to d1, the tMCAO did exhibit significant improvement over the 10 week post-tMCAO period, indicating a significant improvement in function (Figure 4B).

#### Forelimb Cylinder Placement Exhibited Only Transient Change

The tMCAO group exhibited a significant but also transient deficit in forelimb cylinder placement at w1 and w2 post-tMCAO. No other significant differences were observed post-tMCAO (Figure 4C).

### 4. Cognitive Impairment Detected by PA and APA

#### PA Retention Exhibited small change

**Figure 5:**
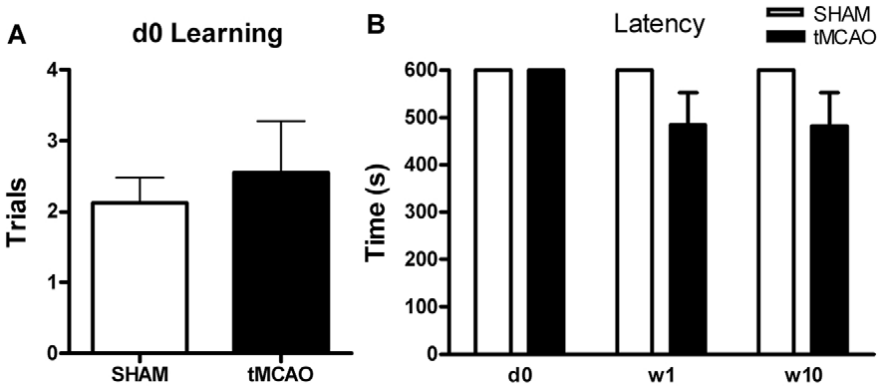
Cognitive performance detected by PA test. **A:** The number of trials to reach learning criteria was similar prior to surgery in both tMCAO and Sham Surgery groups. **B:** The retention of learning by avoiding the dark compartment and shock was measured as the latency in seconds at baseline (d0), w1 and w10 after surgery. Although, there was a trend for tMACO to decrease latency and thus have poorer retention of the learned avoidance with parametric analysis, this was not statistically significant. However, nonparametric analysis of the data did indicate that the sham vs tMCAO group main effect was significant, suggesting overall difference between the two groups, p<0.05. However, the interaction (i.e., Group by Time effect) was not significant (n = 8–9 per group).

A schematic of the PA apparatus is provided in Figure 1A. Before the surgery (d0), the baseline of number of trails to learn (i.e., to reach the criterion of 10 min or 600 s avoidance of entering the dark compartment). There was no difference between tMCAO and Sham groups in the number of trails required to learn to criteria (i.e., to avoid entering the dark compartment for 600 s (Figure 5A). After tMCAO, analyzing the data using ANOVA there was only a trend for decreased latency in tMCAO group to enter the dark chamber (i.e., decreased retention) compared to the sham group. However, using non-parametric analysis there was an overall significant difference on retention between these two groups (i.e., overall group effect in Sham vs tMCAO groups), p<0.05 (see Figure 5B).

#### Robust, Persistent and Progressive Inability to Learn APA

**Figure 6:**
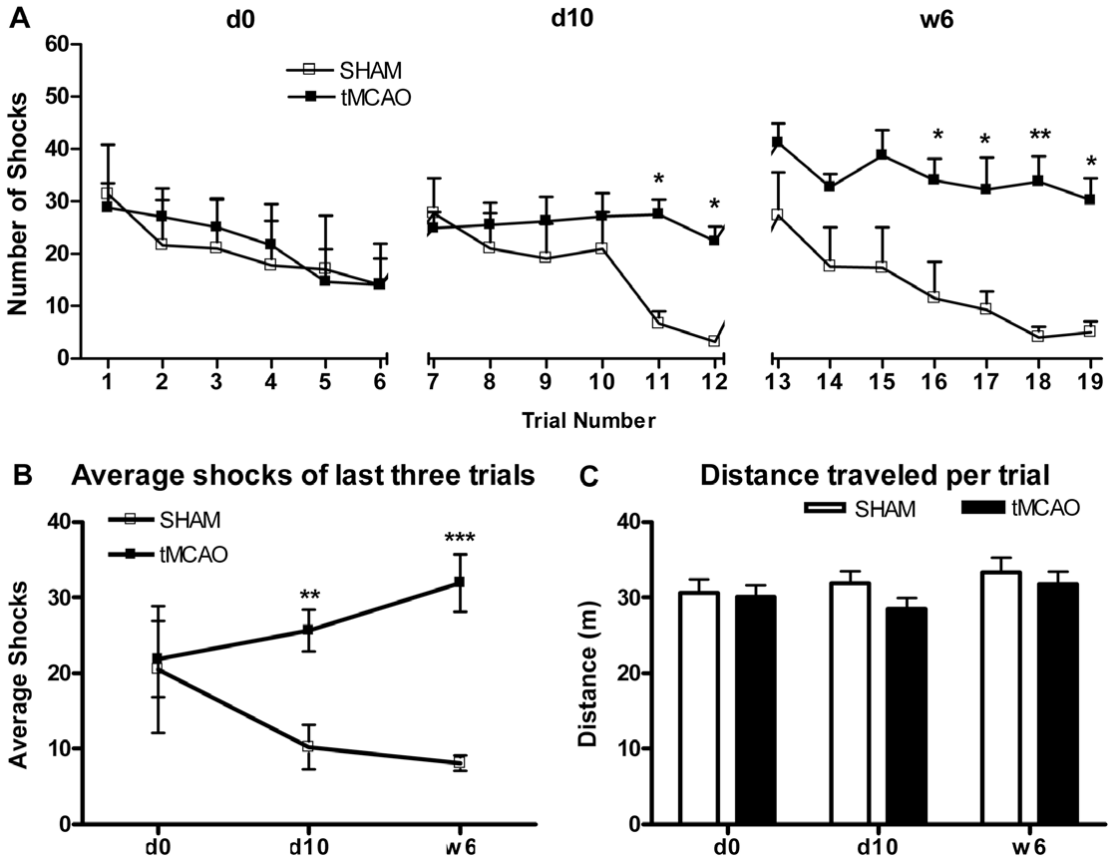
Cognitive performance detected by APA test. **A and B:** Active place avoidance was performed before (d0), and at d10 and in w6 learning sessions following tMCAO or Sham Surgery. In Part B, the number of shocks delivered is presented as a function of each of the six trials during the d0, d10 and w6 learning sessions for Sham vs tMCAO groups. In Part C, the average shock numbers of the last three trials is presented as a function of d0, d10 and w6 learning sessions for Sham vs tMCAO groups. Clearly, the Sham group learned progressively better over learning sessions, while the tMCAO group learned progressively worst over learning sessions. **Part C:** The similar average distance traveled presented as a function of learning sessions indicates that the learning deficit in tMCAO was not due to differences in movement during testing. (*p<0.05, **p<0.01 and ***p<0.001 vs. sham group, n = 8–9 rats/group).

A schematic of the APA apparatus is provided in Figure 1B. Prior to surgery at d0 (i.e., baseline training session), rats were trained in the APA apparatus (i.e., 6 consecutive 10 min trials separated between each by 10 min of rest) and then divided into equally performing Sham and tMCAO groups based on the number of shocks per trial (Figure 6A; d0). Thus, the learning at baseline was identical between the Sham and tMCAO groups. Figure 5B also depicts the results from APA performed at d10 and at w6 following sham or tMCAO surgery. Not only did tMCAO rats exhibit a significant deficit in APA performance at d10 and w6, but they also exhibited a progressive increase in shocks per trial occurring over time from d0 to d10 to w6. Over the same training sessions, the sham group exhibited a progressive improve improvement in APA learning (Figure 6A; d0; d10; w6). When data is presented for the last 3 trials at each training session, the differences between increased improved learning in sham rats compared to the declining learning in tMCAO rats is more clearly depicted (Figure 6B). There was no significant difference in the distance traveled per trial between sham and tMCAO groups during any of the training sessions as depicted at d0, d10 and w6 (Figure 6C). Thus the potential confounding influence of motor functional deficits and/or activity on APA performance in this assay can be excluded. Compared to PA, APA measures more complex cognitive performance. Therefore, APA under these conditions is a more sensitive measurement of post-stroke cognitive impairment, analogous to vascular dementia or vascular cognitive impairment that results from stroke in man

### 5. Infarction Measured By Brain Hemispheric Loss

**Figure 7:**
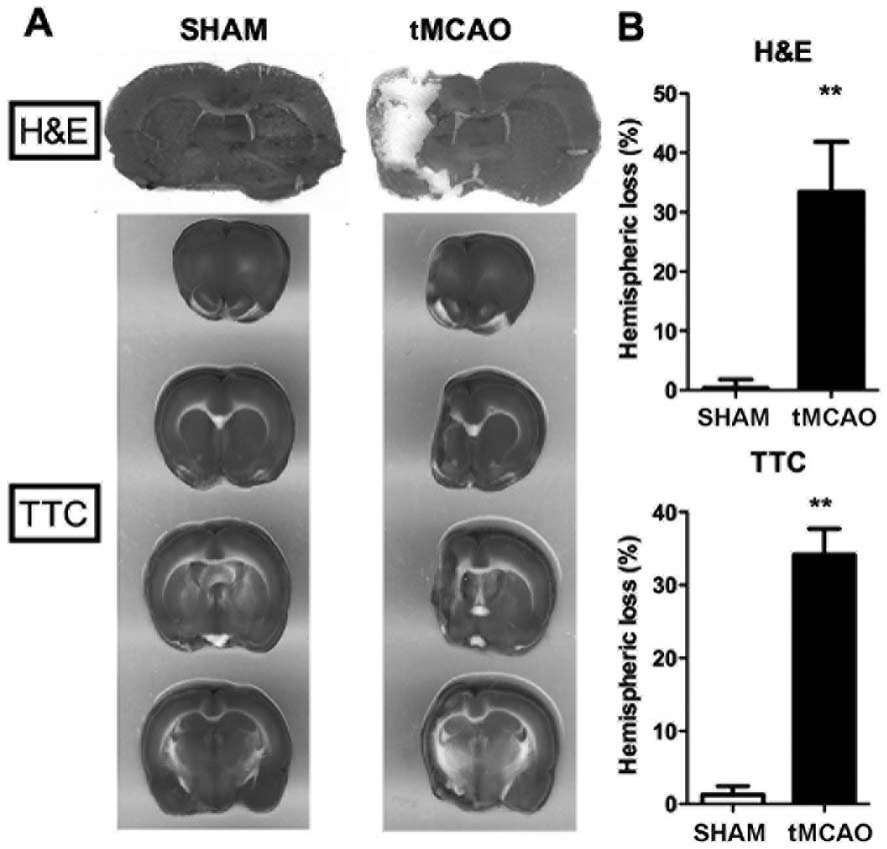
tMCAO produces significant brain hemispheric loss ten weeks after surgery. **A:** Images of coronal forebrain sections show representative H&E and TCC staining in Sham and tMCAO surgery groups. **B:** The percentage of forebrain hemispheric loss reflecting the originally infracted tissue following Sham vs tMCAO surgery is practically identical for the two staining-measurement techniques. **p<0.01 vs. sham group (N = 3–6).

MCAO surgery produced significant brain damage in the ischemic hemisphere as measured by H&E and TCC staining. Injury was measured at termination of the above neurobehavioral determinations (i.e., 10 weeks post Sham or tMCAO surgery). Injury included ischemic hemisphere deformation and hemispheric loss (i.e., due to infracted tissue removal and cavitation). Hemispheric loss of brain sections was measured and reflects the original infarction that occurred following tMCAO as descried previously [27], [28]. Also, the loss of striatum and cortical areas seen in the present study reflects brain injury similar to that described previously by us following tMCAO (Zhou et al, 2011). Practically identical results were observed for the two different stains in thick (2 mm; TTC) and thinner (30 µm; H&E) brain sections (Figure 7A and B), thus validating either technique for future experimental work and as also validate earlier [27], [28].

### 6. Additional Histological Examinations of Post-Surgery Forebrains

Additional histological examination of hippocampus neurons and myelin loss/injury to fiber tracts (i.e., as outlined below) was conducted in groups of rats 4 weeks after Sham or tMCAO surgery. Four weeks post-surgery was chosen because it is sufficiently long after tMCAO to capture all behavioral changes that occurred due to focal ischemic stroke surgery.

#### Hippocampus CA1 Were Unchanged

**Figure 8:**
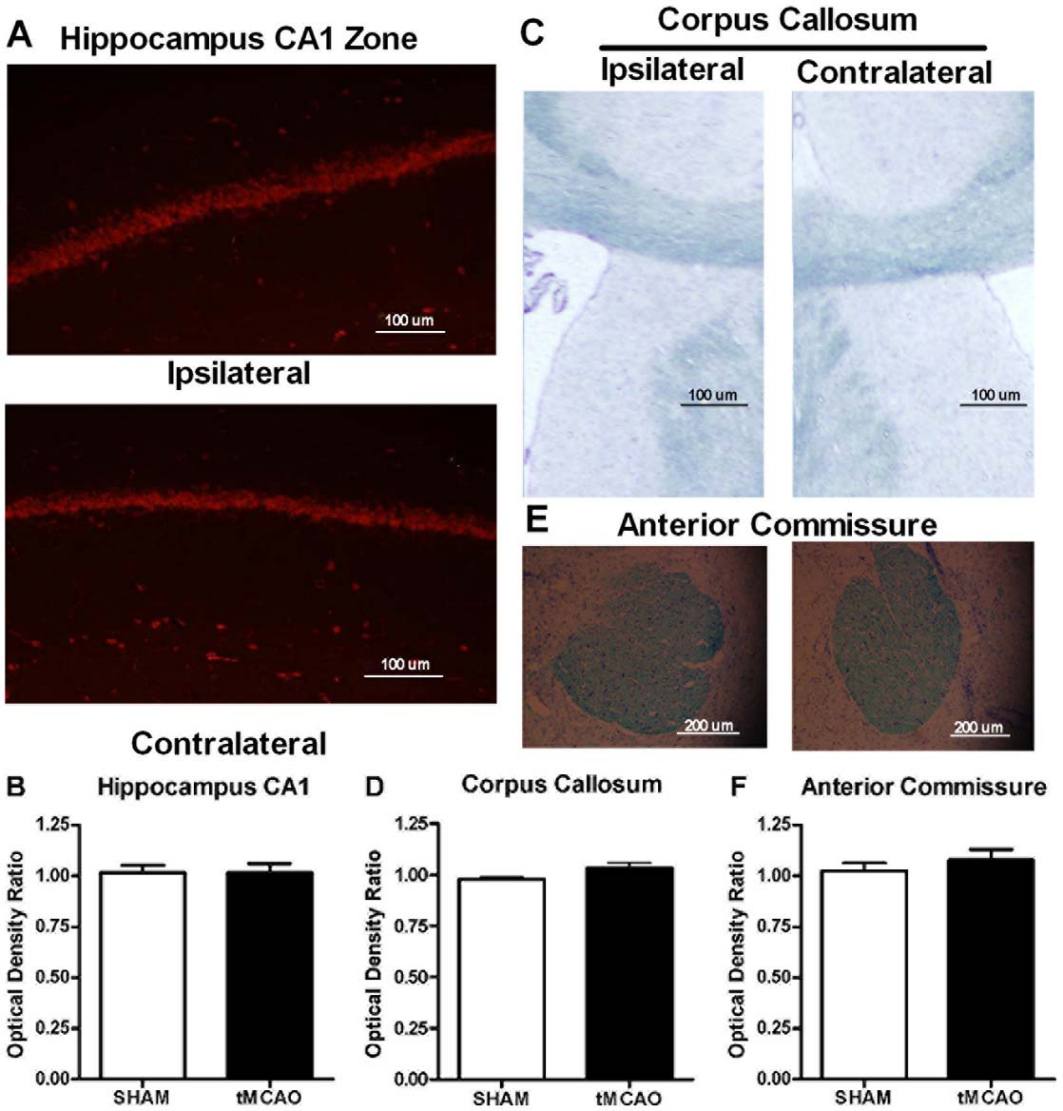
Photomicrographs of forebrain areas and quantified data in graphic form of ischemic ipsilateral vs contralateral control hemispheres of forebrain areas (4 weeks following Sham or tMCAO surgery). **A.** Representative images of NeuN staining in the hippocampus CA1 zone. **B.** As depicted in the images above there were no differences in the optical density ratios between hemispheres for NeuN neuronal cell density of the hippocampus CA1 zone for Sham or tMCAO surgery groups. **C.** Representative images of Luxol Fast Blue myelin fiber staining in corpus callosum. **Part D.** As depicted in the images above there were no differences in the optical density ratios between hemispheres for Luxol Fast Blue myelin fiber staining in the corpus callosum for Sham or tMCAO surgery groups. **Parts E.** Representative images of Luxol Fast Blue myelin fiber staining in anterior commissure. **Part F.** As depicted in the images above there were no differences in the optical density ratios between hemispheres for Luxol Fast Blue myelin fiber staining in the anterior commissure for Sham or tMCAO surgery groups. (N = 3 in Sham group, N = 4 in tMCAO group, student *t* test).

Since hippocampus CA1 neurons are very sensitive to ischemia [33], [34] and are critical to APA performance [26], we measured the CA1 cell layer in sham and tMCAO groups to compare and determine the potential of hippocampus neuronal loss due to focal ischemia. The neural counts were carried out via NeuN staining in the CA1 cell layer. The optical intensity ratio of ipsilateral to contralateral hemisphere was not different between sham or tMCAO groups. Thus, there was no significant CA1 neuronal loss due to tMCAO that could explain the cognitive deficit in APA learning (Figure 8A and B).

#### Myelin in Corpus Callosum and Anterior Commissure

Luxol fast blue-myelin staining was used to evaluate the white matter myelin integrity as an index of fiber tract injury 10 weeks after stroke. Myelin staining in the corpus callosum (Figure 8C and D) and anterior commissure (Figure 8E and F) was not affected by tMACO.

#### Myelin in External Capsule

**Figure 9:**
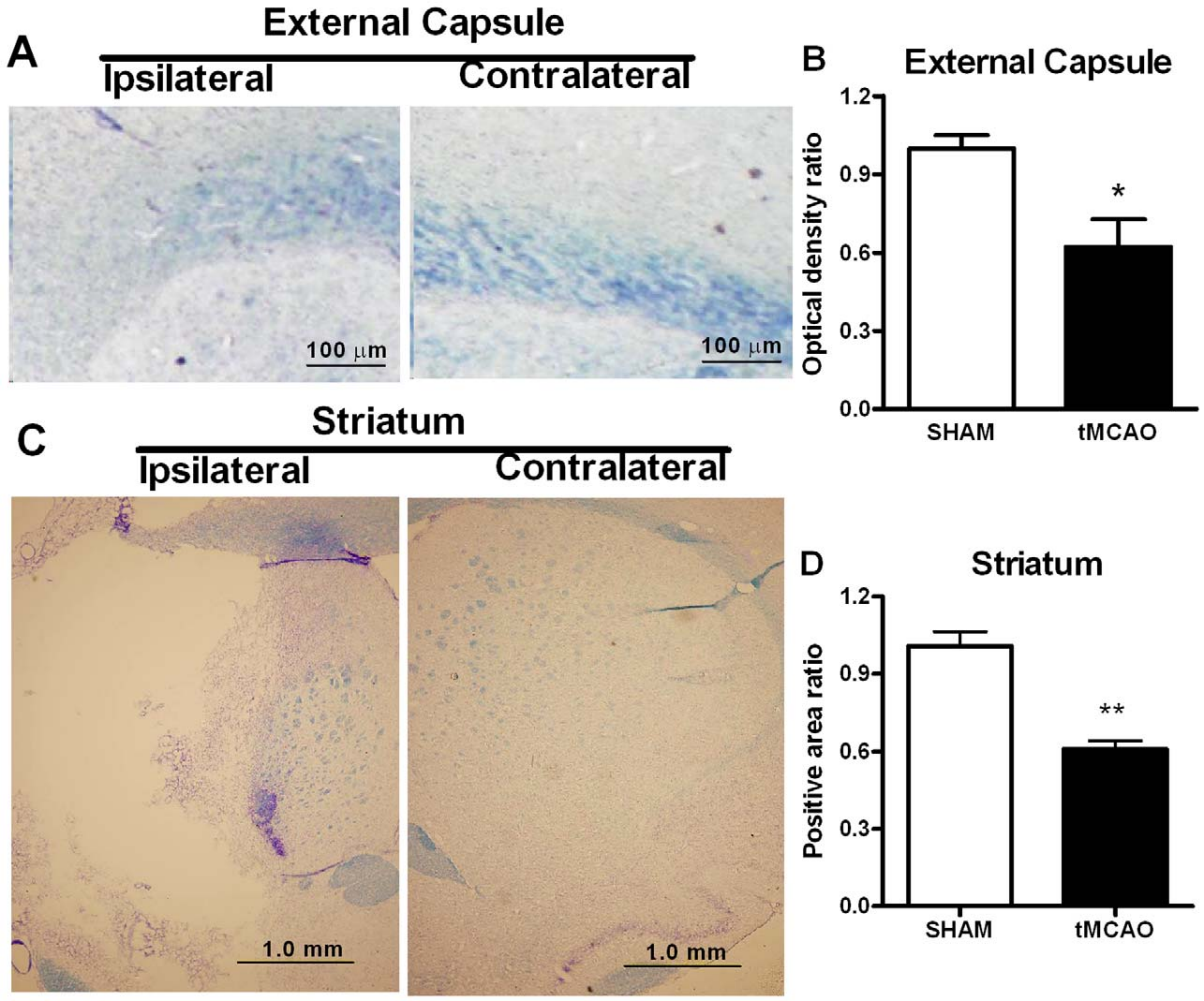
Luxol Fast Blue staining demonstrated myelin loss in the external capsule by attenuated optic density and striatum by decreased positive area for the ischemic ipsilateral compared to the control contralateral hemisphere in tMCAO surgery rats (4 weeks following tMCAO surgery). **A and B:** Images and optical densities presented for the external capsule demonstrating reduced ischemic ipsilateral hemispheric staining following tMCAO surgery that was not seen following Sham surgery. **C and D:** Images and positive areas presented for the striatum demonstrate reduced ipsilateral hemispheric staining due to original striatum injury and tissue loss following tMCAO surgery that was not seen following Sham surgery. (N = 3–4, student t test, *p<0.05 vs sham group).

tMCAO produced a significant myelin loss-white matter injury in the external capsule (i.e., the peri-infarct region). Here myelin staining was decreased (i.e., optical intensity on the ipsilateral side compared to the contralateral side exhibited approximately a 40% decrease) following tMCAO (Figure 9A and B).

#### Myelin in Striatum

In striatum, tMCAO induced in significant decrease in myelin staining-white matter injury. The striatum positive myelin stained area ratio was decreased approximately 65% following tMCAO (Figure 9C and D).

### 7. Retinal Function-ERG Were Unchanged

**Figure 10:**
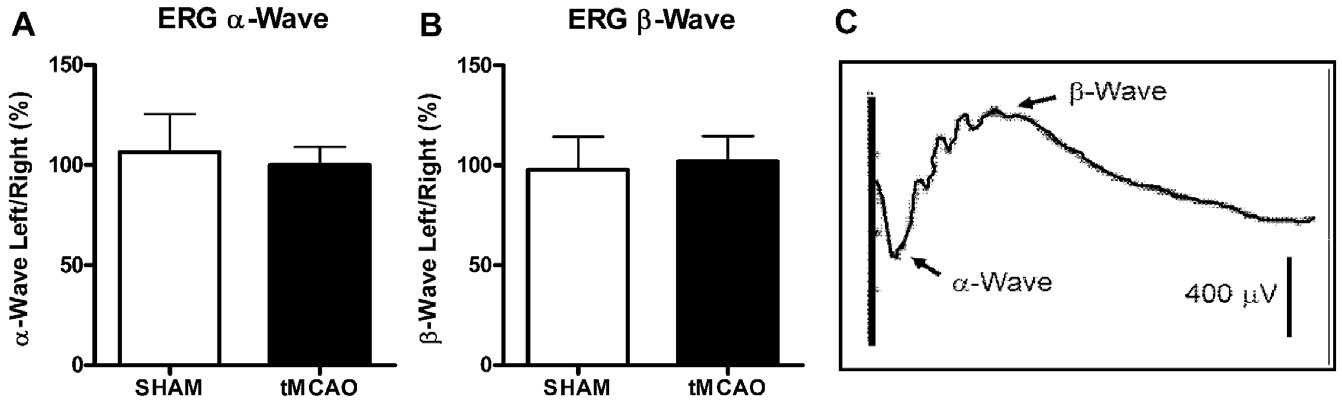
No differences were observed in ERG α-Wave (A) or ERG β-Wave (B) between the ischemic and control side eyes 4 weeks following Sham compared to tMCAO surgery. (N = 7–9, student t test). **C** indicates a representative ERG graph including α- and β-Wave-forms from which the data measurements were made.

ERG was used to measure the effect of tMCAO on retinal function/vision. There was no significant change between the sham and tMCAO groups for either α-Wave or β-Wave amplitudes (Figure 10).

## Discussion

Despite extensive discovery research efforts in stroke, the translational step of moving experimental compounds into the clinic has been met with numerous disappointments [6], [35]. The ultimate goal of the stroke therapy is to improve the life quality for the patients. Hence, the detailed characterization of long term neurobehavioral deficits is critical to experimental stroke research and in the evaluation of the efficacy of therapeutic interventions. Although the demanding task of characterizing sensorimotor and cognitive tasks can provide powerful information on long term deficits and their underlying pathological mechanisms, these parallel data paths for behavior and mechanisms are often lacking [6], [35].

### 1. Long-term and Serial Observation on Neurological Deficits

Longer term stroke studies provide the basis of relatively consistent measurements of brain injury [28], [36] and should provide more definitive information on the persistence of both neurological and cognitive impairments of function [7]. In the present study, we provide a detailed profiling of the long term neurobehavioral changes, including many different neurological and cognitive endpoints, following tMCAO (2 hours occlusion followed by prolonged reperfusion). Tests to evaluate the neurological deficits included: Forelimb foot-fault and cylinder placements and hindlimb placements together with total and individual components of the mNSS [8]. Our profiling of the mNSS is now characterized in detail by separating out the specific component deficits of the mNSS that include: motor, sensory, beam balance and reflexes/abnormal movement responses (Table 1). Thus, a more complete characterization of the long term effects of tMCAO on sensory, motor, proprioception, balance, limb performance, reflexes and movements is now available. Now the outcome evaluations reflecting individually important and complex functions can be extended further to also profile beyond infarct volume changes to those of dendritic structure, synapse number, neurogenesis and angiogenesis changes [37], [38] that better reflect recovery of function in more detail for restoration intervention research in the future. Also, although neurological deficits were most severe immediately (i.e., one day after stroke), persistent changes were identified with many exhibiting trends to decline. As in man, animals in test model systems also display a large degree of spontaneously complete/incomplete recovery within a certain time after experimental cerebral ischemia [39], [40]. Among all the individual neurological functions that were measured, sensory, motor, and beam balance and hindlimb test exhibited the persistent, prolonged deficits indicating that they might be the most sensitive methods for the long lasting changes that need focus for restoration research. It is interesting that forelimb behaviors that involve primarily motor control (i.e., as in foot fault and cylinder placement; Figure 3A and B) were not persistently impaired, but that proprioceptive behaviors of the forelimb (i.e., as in the sensory components of the mNSS; see Table 1 and Figure 2B) and hindlimb (i.e., as in hindlimb placement; see Figure 3A) were very persistent and exhibited no significant spontaneous recovery). Apparently both sensory and motor systems are involved in beam balance where there is an initial large deficit that does decrease dramatically over time but remains significantly impaired for the full 10 weeks of observation. This is apparently related to the focus of cortical brain injury following tMCAO (i.e., in the frontal and parietal regions). In our model, stroke didn't induce any reflex absence and abnormal movements. Also, the short-lived nature of foot fault and cylinder placement also indicate these tests are less important under these conditions. Therefore, it will not be a high priority to continue using these tests in the future.

For many years, different experimental methods in neurological deficits have been developed on animal stroke models, however our research here is different from other studies and has emphasized on the following aspects: 1) We performed a long-term, serial observation, which is not typically performed by other researchers. 2) Instead of a general mNSS as an overall indicator of neurological deficits, we have divided the mNNS into distinct behavioral components (e.g., motor, sensory, beam balance tests and the absence of several reflexes and/or presence of abnormal movementssensory) and investigated each component profile after stroke. 3) As part of this approach, we systematically compared multiple behavioral measurements related to different brain functions and identified the persistence of these deficits (i.e., spontaneous recovery time, etc). Thus, we provide the systematic long term comparisons in functional recovery after stroke. We expect to use these results in our future restorative studies following stroke-induced brain injury and we hope these will help other researchers to link different types of functional deficits to brain protection and recovery.

### 2. Long-term and Comparison of PA with APA Post-stroke

Functional deficits in human stroke are commonly associated not only with impaired sensorimotor ability, but also reduced cognitive function as observed in human vascular dementia or vascular cognitive impairment [41], [42]. In experimental stroke, cognitive testing and assessment of memory functions are evaluated at later time points because they require more normalized motor functions [43], [44]. The two most common assays are the radial arm and water mazes that have been used to assess spatial learning and memory after stroke. However, the radial arm maze requires significant animal training and is very labor intensive in order to reach criterion and the water maze depends largely on intact sensorimotor functions that are altered after stroke. If the impact of sensory or motor deficits is limited, the spatial memory deficits identified from water maze data have been found to be minimal in rodent stroke [45].

In the present work, associative learning and memory has been assessed in experimental stroke using “active place” and “passive place” avoidance paradigms. During the training, the animals actively or passively avoid a part of apparatus where they have learned to associate with receiving an electric shock. Currently avoidance tasks, for example PA, are the most commonly used measures of cognitive function in experimental stroke research [11]. PA is a very simple cognitive paradigm. In the present study PA retention exhibited a statistically significant, but relatively small difference between sham and tMCAO groups. Certainly it is much less robust than the APA learning deficit identified in this work and discussed below.

Using APA, rats on a continuously rotating arena rapidly learn to avoid a mild foot shock by paying attention to their position relative to distal room landmarks. The rats require neither deprivation nor pre-training because the constant rotation of the arena forces the animal to move to a safe position before entering into a previously “punished” shock zone [19]. This method is very dependent on the hippocampus and sensitive to spatial navigation and learning-memory impairments [26]. In this apparatus, the total travel distance during each trial was measured in order to further understand and exclude the influence of altered motor function on the stroke-induced learning deficit. There was never a difference in travel distance between the groups, indicating that this stroke-induced cognitive decline is associated with brain damage early and persistently after stroke, similar to the observations in clinical stroke [41], [42]. Before surgery, there was no statistical difference in the number of shocks between tMCAO and Sham surgery groups, indicating similar learning and memory between the two groups. Thus, any differences that were observed after surgery were due to damage induced by the tMCAO stroke. Compared to PA, we have found that APA is much more sensitive to brain injury and can detect the early, prolonged impairments in cognition after stroke. Cognitive impairment was observed by d10 and was prolonged until w6, demonstrating the long-term learning and memory deficit that can occur following stroke in rats. Indeed, after tMCAO stroke rats are not only cannot learn APA, and even exhibit a progressive increase in this learning deficit over time (i.e., make even more errors over serial sessions). The results here indicated that complex APA performance might be analogous to the decision making required for executive function, and deficits under these conditions might model very well post-stroke changes observed in vascular cognitive impairment/vascular dementia [46]. APA is also very sensitive to traumatic brain injury [16], [47] but has never been applied to experimental stroke research, Also, APA provides a much more sensitive approach to assessing complex cognitive functional impairment post-stroke than the classical use of PA.

### 3. Brain Pathological Changes Including Myelin Loss in Specific Infarct and Peri-Infarct Areas

Experimental cerebral ischemia induces a stress response in neuronal and non-neuronal cells and protection of all cell types must be considered and implemented for brain protection [46]. Although permanent and transient MCAO previously did not cause detectable morphological alterations in the retina or optic nerve, it evokes ischemic stress as revealed by the differential expression of various heat shock proteins in the retina [48]. To evaluate the visual/retinal function after transient focal ischemia in the present work, ERG was used. We found similar ERG α-wave and β-wave responses in both the left and right eye after left tMCAO and absolutely no differences between sham and tMCAO surgery groups at all. Thus, these data demonstrate no effects of tMCAO surgery on retinal or visual function and suggest that the changes in neurological and cognitive functions cannot be associated with visual deficits. Therefore, these data confirm the significance of the brain injuries identified in the present studies to the post-stroke neurobehavioral dysfunctions reported in the present experiments.

In the present studies the occurrences of long-term neurobehavioral dysfunctions were associated with the original brain infarction and later hemispheric tissue loss produced by tMCAO that was confirmed by both H&E staining and TTC staining methods. However, these stains used to measure the degree of brain loss are crude methods to estimate brain injury and, as shown previously, are not necessarily correlated with behavioral outcomes [49]. Therefore, here we decided to explore some more specific staining approaches that have future potential to identify morphological changes related to the present functional changes produced by stroke.

Since (1) cognition, especially APA performance, is highly dependent on an intact hippocampus, and (2) the sensitivity of the hippocampus to ischemic injury is well known, we decided to measure neuronal loss in the highly “ischemia-sensitive” hippocampus CA1 cell layer. We found no significant CA1 cell loss/degeneration after tMCAO. Therefore, under the present conditions hippocampal neuronal cells did not contribute to the cognitive deficits after stroke.

Experimental stroke research, behavioral and histological outcomes do not necessarily correlate well. For example, experimental manipulations that improve behavioral outcomes can occur in the absence of any corresponding change in brain pathology/injury [49], [50]. Of course the most common approach to this measures the amount of histological damage by calculating infarct size following tMCAO [9] or measuring the integrity/density of neurons [51] as above. These methods mainly differentiate the live versus dead tissue/cells, and can't identify diffuse histological changes outside the immediate MCA territory, such as in the peri-infarct area. Moreover, a simply identification of dead cell/tissue can't provide useful information on the cellular function and intercellular communication changes that occur post-stroke. Therefore, much more work to investigate the remaining cellular functioning and intercellular communications need to be directed in the peri-infarct and even remote contralateral hemispheric areas by ourselves and others in the future.

Numerous studies have focused on the role of neurons after stoke. However, the role of myelin has never been equally investigated. White matter injury/lesions have been gaining attention more recently, primarily due their association with cognitive dysfunction [52]. Myelin (i.e., provided by oligodendrocytes) is responsible not only for protection/insulation of axons and proper communication between neurons, but also for production of supportive neurotrophic factors. Thus, loss of myelin will have an adverse effect on axonal function with implicated effects on neuronal communication and multi-cellular survival [53]. The role of myelin in learning and memory functions is likely due to its involvement in information processing such as maintaining connectivity between neuronal circuits important for complex cognitive control and the regulation of conduction velocity regulation [54]. Thus injury to oligodedrocytes and myelin will result in insufficient interconnections of cortical and subcortical regions involved in information processing [55]. In our model, myelin loss was observed in striatum, which is associated with the general tissue loss in this area due to infarction. Moreover, the decreased myelin staining has been observed in external capsule, indicating myelination deficiency did occur in peri-infarct fibers after stroke. Some data on specific fiber systems in cognitive and motor behavior following brain trauma is already available [23], [47]. However, only recently has data become available on axonal connectivity changes associated with post-stroke functional recovery of motor deficits [21]. We are not aware of any data available yet on the involvement of fiber systems in sensory-motor integrative or cognitive behaviors. The effects in the in internal capsule and striatum fiber systems were specific as there were no significant effects of tMCAO on hippocampal neurons, or on white matter injury in the anterior commissure or corpus callosum. Of translational importance, axonal injury in the internal capsule previously has been correlated with motor deficits in patients after stroke [56]. In addition, the loss in the integrity of cortex-striatum white matter pathways has been recently shown to be critical to age-related learning deficits in man [57]. Thus, white matter damage following stroke might contribute to impairments in motor and cognitive information processing within and between hemispheres and in descending motor pathways (e.g., the many functional impairments observed in the present experiments). Clearly other fiber tracts and cell-connections that are disrupted due to the peri-infarct cortical and striatal neuronal loss that occurs following stroke will need to be examined in much greater detail in the future. Larger studies are required that evaluate correlations between specific behavioral and histolopathologic changes in the future. In this way we can identify the variance that can begin to link causative structural and connection injury to more specific behavioral changes. Here we provide only the first investigation into these interactions with more to follow. Clearly we provide information here indicating that the complexity of proper brain functioning extends beyond only simple tissue or cell loss mechanisms. It can be expected to encompass oligodendroglial and also even astroglial loss/dysfunction. These also need more study in the future. Microglial activation in injury/repair also needs to be addressed.

The neurological mechanisms of behavioral deficits after stroke are a complicated process that is not fully understood. In this work we characterized long-term post-stroke brain changes using different histological methods in different brain areas. This will allow brain factors that contribute to long-term stroke functional deficits after stroke are available for use in future work of brain protection and restoration of function. Myelin loss of staining in this study is one of our observations. It is area specific and might be contribute to the neurological and cognitive dysfunction. This is important and suggests that oligodendrocyte injury/impaired regeneration of myelin might be potential targets in post-stroke pathology. A serial evaluation of cellular changes, including various white matter areas including detailed correlation analyses of different neurobehavioral deficits is required in order to help us eventually understand and target the complex mechanisms of restoration of functions post-stroke. The present work has only begun to look at this. Although have established some useful approaches and relationships, it is just the beginning of this journey.

## Conclusions

The present study design provided a comprehensive assessment of neurological and learning-memory measurements post-stroke, including some clear demonstrations of spontaneous recovery/restoration of lost functioning. In summary, the most robust and persistent deficits observed after ischemic stroke were various component neurological-neurobehavioral deficits and the complex, cognitive control required for APA. These data provide valuable, comparative information on appropriate tests useful for studying approaches to induce long-term functional recovery following stroke in future work. Here we showed that APA cognition is an extremely sensitive index of stroke long-term deficits and is relevant to the human condition of vascular dementia-vascular cognitive impairment that can occur post-stroke. Moreover, myelination deficiency in the peri-infarct area appeared to play an important role in cognition impairment after stroke and myelin regrowth mediated by oligodendrocytes should be a focus in future restoration research. These, in addition to diffuse peri-infarct cell loss/protection, changes in angiogenesis and neurogenesis with new synaptic and axonal connections and their protection need to be studied as outcome and mechanism measurements in future experimental stroke research [4].
